# Magnetism of Kesterite Cu_2_ZnSnS_4_ Semiconductor Nanopowders Prepared by Mechanochemically Assisted Synthesis Method

**DOI:** 10.3390/ma13163487

**Published:** 2020-08-07

**Authors:** Katarzyna Lejda, Mariusz Drygaś, Jerzy F. Janik, Jacek Szczytko, Andrzej Twardowski, Zbigniew Olejniczak

**Affiliations:** 1 Faculty of Energy and Fuels, AGH University of Science and Technology, Mickiewicza 30, 30-059 Krakow, Poland; kkapusta@agh.edu.pl (K.L.); madrygas@agh.edu.pl (M.D.); 2 Faculty of Physics, Institute of Experimental Physics, University of Warsaw, Pasteura 2, 02-093 Warszawa, Poland; Jacek.Szczytko@fuw.edu.pl; 3 Institute of Nuclear Physics, Polish Academy of Sciences; Radzikowskiego 152, 31-342 Krakow, Poland; Zbigniew.Olejniczak@ifj.edu.pl

**Keywords:** kesterite Cu_2_ZnSnS_4_, mechanochemistry, solid-state NMR, SQUID, magnetic properties, oxidation

## Abstract

High energy ball milling is used to make first the quaternary sulfide Cu_2_ZnSnS_4_ raw nanopowders from two different precursor systems. The mechanochemical reactions in this step afford cubic pre-kesterite with defunct semiconducting properties and showing no solid-state ^65^Cu and ^119^Sn MAS NMR spectra. In the second step, each of the milled raw materials is annealed at 500 and 550 °C under argon to result in tetragonal kesterite nanopowders with the anticipated UV-Vis-determined energy band gap and qualitatively correct NMR characteristics. The magnetic properties of all materials are measured with SQUID magnetometer and confirm the pre-kesterite samples to show typical paramagnetism with a weak ferromagnetic component whereas all the kesterite samples to exhibit only paramagnetism of relatively decreased magnitude. Upon conditioning in ambient air for 3 months, a pronounced increase of paramagnetism is observed in all materials. Correlations between the magnetic and spectroscopic properties of the nanopowders including impact of oxidation are discussed. The magnetic measurements coupled with NMR spectroscopy appear to be indispensable for comprehensive kesterite evaluation.

## 1. Introduction

The quaternary sulfide Cu^(1+)^_2_Zn^(2+)^Sn^(4+)^S^(2−)^_4_ called in short kesterite or CZTS has been a focus of intense research work for more than a decade now not only because of its great potentials in the next generation photovoltaics [1,2,3,4,5], but also because of its very much elusive chemical and physical characteristics. The latter aspect stems mainly from the compound’s capability to exist in stable forms with significant non-stoichiometry (e.g., copper rich or copper poor), with variable/interchangeable atom sites in the crystal lattice (e.g., Cu/Zn ordered or disordered polymorphs, kesterite vs. stannite structures), and relatively easily accommodating various structural defects (e.g., vacancies, nanophase segregation) to name the most outstanding [6,7,8,9,10,11,12]. It is also worth pointing out that the chemistry leading to the quaternary kesterite is by the very nature complex and often leads to detectable amounts of post-synthesis impurities or by-products including the relevant binary and ternary metal sulfides [13,14,15,16]. All this seems to effect investigations of kesterite by way of making them inadvertently more of the case than clear-cut fundamental studies of the still poorly reproducible syntheses. Not to mention a common inattention given to post-synthesis gas adsorption and plausible surface-to-bulk oxidation processes upon nanocrystalline kesterite exposure to air.

The interaction of sulfur *2p*-electrons with copper *3d*-orbitals, as theoretically predicted and observed in copper sulfides [17,18,19], may add to the covalent characteristics of Cu-S bonds and make the oxidation states of all the elements, including sulfur, in kesterite merely formal to meet the rules of charge neutrality. It is instructive to realize that a series of copper sulfides of stoichiometries between those corresponding to Cu_2_S (formally Cu^1+^ with diamagnetic configuration [Ar]3d^10^) and CuS (formally Cu^2+^ with paramagnetic configuration [Ar]3d^9^) all appear to contain only Cu^1+^ ions in the solid state [17]. The overall formula Cu_3_S_3_ such as detailed in (Cu^1+^)_3_(S_2_^2^^−^)(S^1^^−^) and (Cu^1+^)_3_(S_2_^1^^−^)(S^2^^−^), with the two latter formulae including various sulfur ions in different S-oxidation states, was proposed to visualize this for mineral covellite CuS [18]. In fact, in many solid-state compounds Cu^1+^ may not be purely *3d^10^* and Cu^2+^ may not be *3d^9^* but these oxidation states should be considered as having a different degree of *d*-orbital occupancy with Cu^1+^ being just closer to the diamagnetic configuration *3d^10^*. All this is also a manifestation of the flexible ionic and structural roles of the S-counter-ions with diverse composition and various oxidation states.

The interaction of possible electronic *d*-orbital paramagnetism with magnetic field of nuclei will contribute to local field modifications and consequently have a definite impact on nuclei shielding and effective magnetic fields in the kesterite crystal lattice. Since a nanocrystallite can be visualized as having a significant share of atoms in the likely disordered surface compared to a more ordered core, the relevant lattice domains will exhibit different symmetries and, therefore, varying nuclear magnetic resonance conditions. It is probable that depending on the strength of paramagnetism coupled with the characteristics of lattice disorder, some nuclei may, and some may not fulfill nuclear magnetic resonance conditions—not all nuclei could, therefore, be seen for this reason by NMR. This has to be confronted with the observation that in the copper sulfide Cu_2_S with the non-magnetic Cu^1+^ and S^2−^ ions no solid-state ^63^Cu NMR resonance could be detected at all [19]. It was proposed that the Cu^1+^-ion low symmetry local environment and very large quadrupolar coupling constants resulted in significant second order quadrupolar interactions that broadened the resonance signal beyond detection. It appears that in the structurally and electronically complex kesterite nanopowders the paramagnetic factor could be entangled in a complex way with nuclear magnetic resonance output, at the end both having relevance to the crucial kesterite’s semiconductor properties.

Given the above observations and since one of the potential metal centers in kesterite is *d*-orbital magnetic, i.e., Cu^2+^ with the formal *3d^9^* configuration while the expected copper sites are nominally Cu^1+^ with diamagnetic configuration *3d^10^*, kesterites’s magnetic properties may come to play a significant role in defining its fundamentals—under certain stoichiometry and synthesis conditions these two types of copper centers can coexist in the kesterite’s lattice. In addition, the high propensity of kesterite to accommodate variable element stoichiometries, extended site occupancy/disorder features, and various defects makes its nanocrystalline forms the firm candidates for the so-called *d^0^* magnetism observed in many disordered/defected nanomaterials including formally diamagnetic zinc sulfide ZnS [20,21,22]. Therefore, the combination of nuclear magnetic resonance measurements sensitive to close range ordering and local field peculiarities with measurements of overall magnetism reflecting averaged magnetic fields may yield a comprehensive insight into the characteristics of kesterite.

In our initial study of the two-step mechanochemically assisted synthesis of kesterite nanopowders from the constituent elements according to the overall reaction 2Cu + Zn + Sn + 4S → Cu_2_ZnSnS_4_, the diverse magnetic and spectroscopic properties of the raw and annealed final products were observed [23]. The raw product from the high-energy ball milling first step, which had a Cu_2_ZnSnS_4_ stoichiometry and a cubic crystal structure (tentatively called pre-kesterite), showed no semiconductor properties and produced no ^65^Cu and ^119^Sn MAS NMR spectra whereas exhibiting clearly paramagnetic properties confirmed by EPR measurements. On the other hand, such a nanopowder annealed under argon at increased temperatures, preferably, of the order of 500 °C (tetragonal kesterite) displayed the semiconductor behavior by UV-Vis spectroscopy and the anticipated NMR characteristics with simultaneously decreased yet still present paramagnetism. Additionally, the XPS measurements showed no evidence of magnetic Cu^2+^ ions in any of the materials pointing out to other sources of magnetism than purely *d^9^* Cu^2+^-ion configuration. The XRD patterns clearly confirmed only one crystalline product in each case while the lowest detectable contents of possible nanocrystalline by-products were estimated by us to be of the order of *circa* 1 wt.%. Therefore, some minor by-products in the nanosized range or those highly amorphous could have gone undetected. Based on these circumstances, well focused and sensitive magnetic measurements such as provided by a SQUID magnetometer for an extended pool of kesterite products seemed indispensable to address the question of kesterite’s magnetism. Also in our view, the aforementioned NMR characteristics can be interrelated with the kesterite bulk magnetism and provide information pertaining to the subject.

Based on the discussed reports and our earlier work describing kesterite synthesis, herein, a comprehensive study is reported of kesterite nanopowders synthesized with a mechanochemically assisted method from two precursor systems, i.e., (i) composed of the constituent elements 2Cu + Zn + Sn + 4S and (ii) of the metal sulfides and sulfur (first time reported precursor system of Cu_2_S + ZnS + SnS + S), including effects of nanopowder long-time conditioning in air. The use of two different precursor systems is aimed at avoiding a case-study syndrome and provide with a broader range of results for the appraisal of kesterite magnetism. In order to exclude/minimize effects of metal site non-stoichiometry on the probable adventitious formation of magnetic centers Cu^2+^, stoichiometric quantities of metals were applied. It is worth to note that the high energy ball milling is confined to the closed reaction space of the grinding bowl and, therefore, creates favorable conditions in this step for completion of substrate reactions in both precursor systems with no circumstantial reagent and by-product losses. The magnetic properties of all nanopowders were determined with a SQUID magnetometer, the close-range nuclear magnetic resonance characteristics were derived with solid-state ^65^Cu and ^119^Sn MAS NMR, energy states of elements were analyzed by XPS technique, whereas the crucial semiconductor characteristics were probed with UV-Vis spectroscopy.

## 2. Experimental

**Synthesis. Step 1–high energy ball milling.** Stoichiometric amounts of commercially available powders (typically, 2 at.% excess of S), i.e., for (i) constituent element system (CE)—with the 2:1:1:4 mole ratio of copper Cu, zinc Zn, tin Sn, and sulfur S and for (ii) metal sulfide system (MS)—with the 1:1:1:1 mole ratio of copper sulfide Cu_2_S, zinc sulfide ZnS, tin sulfide SnS, and sulfur S, for each system totaling *circa* 7 g, were placed in the 20 mL grinding bowl of planetary micro mill Pulverisette 7 (Fritsch) together with standard 80 tungsten carbide WC balls (5 mm) and 6–7 mL of xylene as a dispersion fluid. Upon preliminary trials, the effective milling times were either 4 h for the CE system or 20 h for the MS system, which were realized by 3-min milling cycles each followed by a 10-min idle period to prevent from bowl overheating. The rotation speed was 900 (MS) or 1000 rpm (CE); the latter was the highest achievable speed with the applied set of the bowl and balls and enabled complete reactions in a relatively shorter time of 4 h. After milling, the bulk of xylene was evaporated/dried at room temperature and the resulting still “wet” black solid was transferred to a flask to be further evacuated for 1 h affording a nanopowder of raw product (pre-kesterite). **Step 2–annealing under argon atmosphere**. After sampling the raw product for characterization, it was subjected to annealing in an alumina crucible for 6 h under an argon flow of 0.05 L/min at 500 or 550 °C. The off-black product was slowly cooled to room temperature and later evacuated for 0.5 h to afford the target nanopowder material (kesterite).

**Sample labeling.** Three freshly made target products from each precursor system (constituent element system–CE and metal sulfide system–MS), i.e., pre-kesterite, kesterite annealed at 500 °C, and kesterite annealed at 550 °C will be referred to the abbreviated form of the original system. For instance, pre-kesterite from MS system, 500 °C-kesterite from MS system, and 550 °C-kesterite from MS system. For the three products from the MS system, which were additionally exposed/oxidized in ambient air for 3 months, a suffix OX will be used as in pre-kesterite_OX, etc. 

**Characterization.** All powders were routinely characterized by powder XRD analysis (Empyrean PANalytical, Cu K_α_ source; 2Θ = 10–110°) with average crystallite sizes D_av_ evaluated from Scherrer’s equation applying the Rietveld refinement method [24]. For the purpose of this study, the lattice constants were rounded up to the nearest 0.001 Å and D_av_ to the nearest 1 nm. Selected materials were investigated by photoelectron spectroscopy XPS (Vacuum Systems Workshop Ltd., Crowborough, UK, Mg anode with photon energy of 1253.6 eV) [25]. Magnetization measurements for materials in function of magnetic field (0–7 tesla) and temperature (2–300 K) were carried out using a SQUID magnetometer [26]. The mass-maximized for high intensity signal powders were placed in gelatin capsules showing controlled diamagnetic signal. Solid-state MAS NMR spectra [27] were measured on the APOLLO console (Tecmag), at the magnetic field of 7.05 tesla produced by the 300 MHz/89 mm superconducting magnet (Magnex). A Bruker HP-WB high-speed MAS probe equipped with the 4 mm zirconia rotor and KEL-F cap was used for sample spinning. The ^65^Cu MAS NMR spectra were measured at 85.11 MHz using a single 2 μs rf pulse corresponding to π/4 flipping angle in the liquid. The spinning speed was 6 kHz. The acquisition delay used in accumulation was equal to 10 s and 256 scans were acquired. The peak position scale in ppm was referenced to the ^65^Cu resonance of CuCl—for quadrupolar ^65^Cu nuclei, this scale is not identical with the chemical shift scale (uncorrected for second-order quadrupolar interactions). The ^119^Sn MAS NMR spectra were measured at 111.68 MHz using a single 3 μs rf pulse corresponding to π/2 flipping angle. The spinning speed was 6 kHz. The acquisition delay used in accumulation was 30 s and 256 scans were acquired. The peak position scale in ppm was referenced to the central transition of SnS spectrum located at −299 ppm—for ^119^Sn nuclei, this scale is identical with the chemical shift scale. Both ^65^Cu and ^119^Sn spectra were mass-normalized per 1 g of sample. UV-Vis data [28] were collected on a Perkin-Elmer spectrophotometer Lambda 35 equipped with a 50 mm integrating sphere for direct measurements of powder samples.

## 3. Results and Discussion

**(a) Synthesis, structure, and spectroscopic properties.** In this project, we used the mechanochemically assisted synthesis of kesterite starting from two precursor systems, i.e., (i) previously explored by us [23] and others [29,30,31] mixture of the constituent elements Cu, Zn, Sn, and S (CE system), presently, at the highest achievable rotation speed of 1000 rpm, and (ii) a mixture of the selected metal sulfides Cu_2_S, ZnS, SnS, and supplementary sulfur S (MS system), which to the best of our knowledge is reported for the first time. In the former case, a preferential formation of covellite CuS was expected to occur in the initial stages of reactions since merely hand-grinding such a mixture was found by us in separate experiments to produce it. This would set the stage for subsequent redox reactions in which not only remaining sulfur S^0^ but also various S-ions present in CuS would participate [18]. In the case of the MS system reacting overall according to Cu_2_S + ZnS + SnS + S → Cu_2_ZnSnS_4_, the prevailing redox chemistry should involve, in the first approximation, only tin (oxidation from Sn^2+^ to Sn^4+^) and sulfur (reduction from S^0^ to S^2−^) making it from that standpoint simpler. Owing to the application of two precursor systems/synthesis routes and two annealing temperatures of 500 and 550 °C in the optimum pyrolysis range for kesterite, the resulting nanopowders represent a diverse pool of materials for further studies.

**Figure 1:**
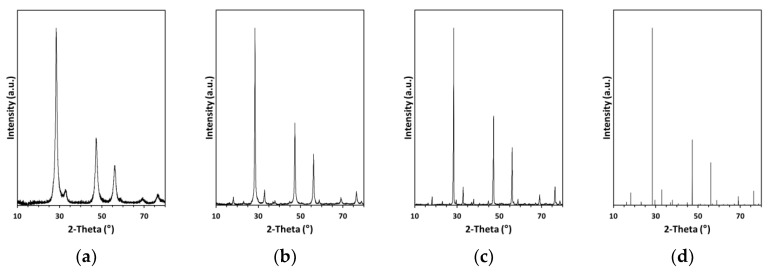
XRD patterns for nanopowders in the metal sulfide (MS) precursor system: (**a**) freshly milled pre-kesterite; (**b**) 500 °C-kesterite; (**c**) 550 °C-kesterite; (**d**) bar chart for tetragonal (I−42m) kesterite.

The XRD patterns for the nanopowders in the MS system are shown in Figure 1 and they can be satisfactorily compared with the respective patterns in the CE system as previously reported by us [23]. The patterns for the milled/fresh nanopowders in both systems confirm a single crystalline product of cubic symmetry typical for pre-kesterite with the average crystallite size D_av_ determined from Scherrer’s equation, i.e., for CE: a = 5.518 Å, D_av_ = 10 nm and for MS: a = 5.424 Å, D_av_ = 10 nm.

Annealing of the pre-kesterite at elevated temperatures produced the expected kesterite of tetragonal symmetry in the space group I−42m and of comparable lattice parameters, i.e., for CE at 500 and 550 °C: a = 5.432 Å, c = 10.830 Å and a = 5.433 Å, c = 10.837 Å, respectively, and for MS at 500 and 550 °C: a = 5.417 Å, c = 10.880 Å and a = 5.432 Å, c = 10.837 Å, respectively. Also, the higher annealing temperature clearly favored more efficient crystal growth and resulted in larger average crystallite sizes as seen by comparison of annealing at 500 and 550 °C in the CE system (D_av_ of 35 vs. 90 nm) and in the MS system (D_av_ of 20 vs. 50 nm). These results confirm the same products of the high-energy ball milling and annealing stages in both systems and fully support our originally reported findings. However, the small while significant variations in the lattice and crystallite size parameters point out to some specific features of each substrate system.

**Figure 2:**
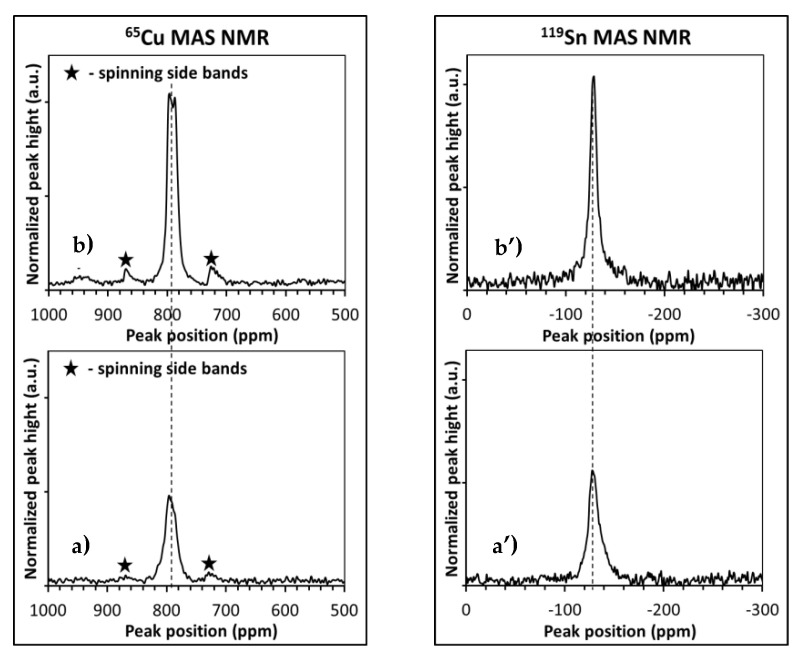
Mass-normalized solid-state ^65^Cu (left) and ^119^Sn (right) MAS NMR spectra for kesterite samples in MS system (Cu_2_S + ZnS + SnS + S) annealed at 500 °C (**a**,**a’**) and 550 °C (**b**,**b’**). Dotted lines in peak positions are guides for eye, only.

For both raw nanopowders made after milling in the two precursor systems, no solid-state ^65^Cu/^119^ Sn MAS NMR spectra could be obtained as anticipated for pre-kesterite. The spectra could, however, be recorded for the annealed samples as exemplified in Figure 2 for the MS system (see, [23] for the CE system). The peaks for the 500 °C-annealed kesterite are broader and less intense (bottom row) than those for the 550 °C-annealed product (top row) differing only slightly if at all in peak positions. The latter peaks are typical for well crystallized materials, i.e., the ^65^Cu MAS NMR peak at *circa* 792 ppm has a characteristic “tower” shape for quadrupolar copper nuclei with spin 3/2 and the ^119^Sn NMR peak at *circa* –128 ppm is relatively narrow and symmetrical as expected for isotropic tin nuclei with spin 1/2, both features indicating a well-defined close range environment for either of the metal nuclei. This is despite two non-equivalent/distinguishable copper sites in tetragonal kesterite. On the other hand, the 500 °C-annealed kesterite data are indicative of a less homogeneous environment—the copper resonance broadens and loses its fine structure while the tin resonance broadens and, additionally, becomes asymmetric upfield consistent with a wide and inhomogeneous distribution of chemical shifts for a proportion of Sn nuclei [12]. What strikes is a pronounced difference in mass-normalized peak intensities between the 500 °C- and 550 °C-annealed powders true for both metal nuclei, i.e., the peaks for the 500 °C-nanopowder are relatively much less intense. A calibration ^65^Cu MAS NMR experiment for a solid mixture of known amounts of the 550 °C-annealed kesterite and the reference CuCl was performed for a quantitative spin estimation. This was realized by calculating a normalized amount of resonant ^65^Cu nuclei in kesterite with the theoretical formula Cu_2_ZnSnS_4_ from comparison of both signal intensities assuming that all ^65^Cu nuclei resonate in CuCl. Based on relative peak intensities, this enabled to get such an estimation also for the 500 °C-annealed kesterite. Surprisingly, the calculations provided for the 550 °C- and 500 °C-nanopowders with mere 24% and 12% of all nuclei, respectively, to be active in the NMR experiment. In this regard, our Cu/Zn-site disordered kesterite (space group I−42m) has symmetry differentiated Cu nuclei in the *2a* and *2c* sites as supported by the relevant ^65^Cu static NMR experiments yielding the respective narrow and wide band [12]. The copper resonance in our spectra may be accounted for by the fact that in the current ^65^Cu MAS NMR experiments the tensor characteristics of the wide band could have resulted in the relevant subset of Cu nuclei being undetected. However, even taking this factor into account, there is still a significant share of Cu nuclei in the kesterite not active/probed in the ^65^Cu MAS NMR measurements. This could be due to emergence of lattice domains with Cu^1+^-ion low symmetry local environment and resulting very large quadrupolar coupling constants, i.e., similar circumstances as invoked in the already discussed case of no ^63^Cu NMR spectra recorded for Cu_2_S [19]. Such domains could be associated, for example, with the surface (more disordered with lower symmetry) vs. core (better ordered with higher symmetry) structure differences in nanocrystallites. But, more likely this could also be linked to specific short-range interactions with kesterite’s locally scattered magnetic centers. In such a case, the ^65^Cu MAS NMR peak intensity trend should be paralleled by the ^119^Sn MAS NMR peak intensities, which in fact is observed—there is a similar impact of annealing on NMR resonance conditions for both the Cu and Sn nuclei.

**Figure 3:**
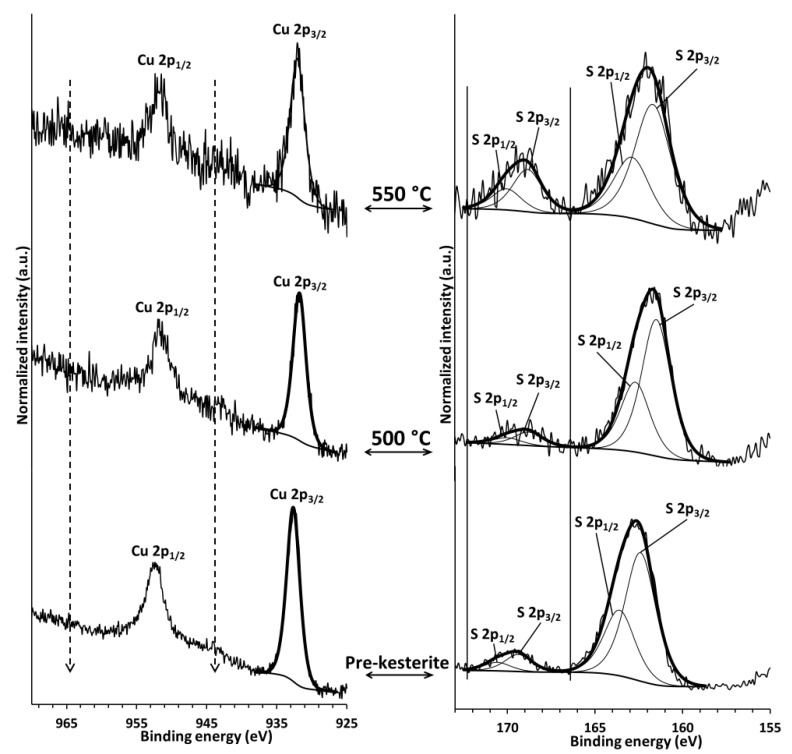
Copper Cu 2p (left column) and sulfur S 2p (right column) XPS signals for nanopowders in the metal sulfide (MS) system: bottom—raw powder of pre-kesterite, middle—kesterite after annealing at 500 °C, top—kesterite after annealing at 550 °C. Dash lines in left column are eye-guides for Cu^2+^ shake-up band approximate positions. Solid lines in right column show the region of S 2p band characteristic for S–O bonds.

The X-ray photoelectron spectroscopy (XPS) is considered to be a surface-sensitive technique analyzing characteristic photon energies of elements emitted from a material’s surface layer (up to 10 nm deep) upon X-ray irradiation [25]. For many nanopowders, though, the analysis is bulk averaged out for both nanocrystallite surfaces and cores. Figure 3 represents typical in this study Cu 2p and S 2p spectra for the pre-kesterite and both annealed kesterite nanopowders shown for the MS system. In the left column, a characteristic doublet of the Cu 2p_1/2_ and Cu 2p_3/2_ bands/peaks is seen for each material, which in the standard way is reported as a position of the higher intensity/lower energy peak Cu 2p_3/2_. The latter positions are in the range 931.9–932.2 eV typical for various Cu^1+^-ion containing materials [32,33] including kesterite [34,35]. For instance, in anhydrous Cu^(2+)^SO_4_, this peak is found at 936 eV being part of the Cu 2p doublet located on the high energy side of the doublet in the Cu^1+^-compounds. A characteristic feature of the Cu^2+^-ion 2p doublet is an associated doublet of shake-up peaks, quite intense and broad, in the positions indicated by the dash lines in Figure 3. It is apparent that no such features are present in any of the nanopowders consistent with no significant quantities of Cu^2+^ ions. With regard to the Zn 2p and Sn 3d results (not shown), they clearly agree with the sulfide nature of these metal ions in kesterite. In the right column, the S 2p peaks are shown for the materials. The higher intensity band at lower energy is actually a doublet of S 2p_1/2_ and S 2p_3/2_ peaks for kesterite with the S 2p_3/2_ peak position in the range 161.5–162.2 eV as confirmed also by other reports [34,35]. Interestingly, there is yet another band at higher energies seen for all the nanopowders which can be de-convoluted into a doublet with the S 2p_3/2_ peak position in the range 168.8–169.2 eV (Figure 3, solid lines in right column). This is a band characteristic for various inorganic sulfate materials, generally, S–O bonds [33] and tenaciously suggests some kesterite oxidation—a mostly overlooked aspect of kesterite properties. Finally, the XPS measurements for the nanopowders from the CE synthesis route provide the qualitatively matching results.

**Figure 4:**
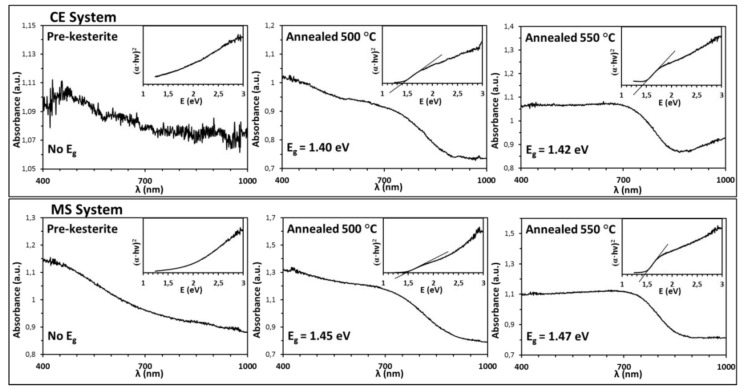
UV-Vis spectra with inserts of Tauc (αhν)^2^ vs. hν (energy) plots and calculated energy band gaps E_g_ for pre-kesterite (left) and annealed at 500 °C (middle) and 550 °C (right) kesterite nanopowders: top row—constituent element (CE) system, bottom row—metal sulfide (MS) system.

The energy band gap E_g_ is the essential property of kesterite in photovoltaic applications that, generally, aim for maximum efficiency at E_g_’s of sunlight absorbers in the range 1.2–1.4 eV [36]. The reported for various kesterite materials E_g_ values span from 1.0 to 1.5 eV making kesterite a prime candidate here. The band gap is conveniently estimated from UV-Vis measurements upon application of the Kubelka-Munk transformation via Tauc’s function, being derived by plotting a tangent curve and recording its intersection with the energy axis as shown in Figure 4. It contains the UV-Vis spectra with inserts of Tauc’s plots for the pre-kesterite and annealed kesterite nanopowders prepared via both synthesis routes. The most important observation is that both pre-kesterite samples are shown to have non-specific absorption and no energy band gap can, therefore, be calculated based on the standard UV-Vis workup of the spectra. This is in agreement with all investigated by us to-date systems applying the high energy ball milling [23,37]. The annealing at 500 °C and 550 °C changes this dramatically and differs only slightly for the two precursor systems. For the CE system, the absorption spectra are quite similar to each other and give comparable E_g_ values in the range 1.40–1.42 eV whereas for the MS system the spectra vary a bit yielding slightly higher E_g_ values of 1.45–47 eV. The absorption curve shape differences can likely be traced to varying size distributions of agglomerated particles in the microsized range and accompanying non-specific light scattering effects. The essential observation is that the annealing results in transformations of the non-semiconducting pre-kesterite to kesterite semiconductor with the band gap in the range 1.4–1.5 eV that slightly depends on the applied precursor route. This evolution of semiconductor properties is accompanied by the phase change from the cubic to the tetragonal polytype as evidenced by XRD but, also, by a strikingly different NMR response.

**(b) Magnetic study.** Magnetization as a function of temperature (2–300 K) and magnetic field (0–7 tesla) was measured with a SQUID magnetometer for all materials from the two systems, specifically, for such samples that after preparation were stored under vacuum in sealed glass ampoules to be fresh-opened before taking measurements. Additionally, for the MS system all powders after the initial measurements were exposed to ambient air for 3 months and measured again to yield rare time-related data on magnetization. In this regard, any oxidation of the initial diamagnetic Cu^1+^ in pre-kesterite/kesterite to magnetic Cu^2+^ would have had a pronounced effect on sample magnetism and, possibly, other properties as well. At this point, it is worth to recapitulate that each precursor system series of the related kesterite materials with the same chemical formula Cu_2_ZnSnS_4_ is made of nanopowders characterized both by the distinct crystallographic structure and specific average crystallite size diameter D_av_, i.e., for MS system: pre-kesterite (cubic, 10 nm), 500 °C-kesterite (tetragonal, 20 nm), 550 °C-kesterite (tetragonal, 50 nm) and for CE system: pre-kesterite (cubic, 10 nm), 500 °C-kesterite (tetragonal, 35 nm), 550 °C-kesterite (tetragonal, 90 nm). It is also worth to note that the thermally induced conversion of the pre-kesterite to kesterite is associated with the change of structure and with crystallite growth that together reflect significant reorganization on the atomic scale. But also, different annealing temperatures yield structurally identical kesterite materials with varying average crystallite diameters and specific surface area/reactivity, i.e., 550 °C-kesterite is clearly better crystalized and expected to be less reactive than its 500 °C-kesterite counterpart.

**Figure 5:**
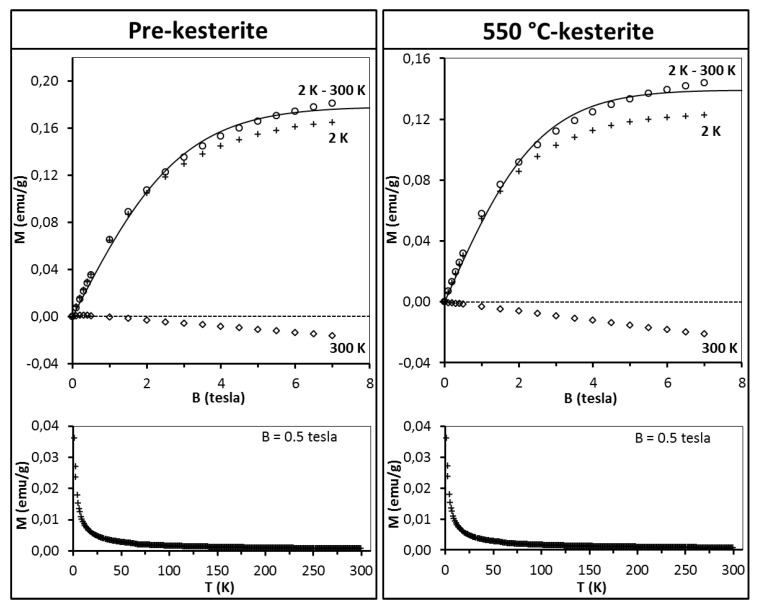
Magnetization M of pre-kesterite (left) and 550 °C-kesterite nanopowders (right) from metal sulfide (MS) system. Upper drawings—magnetization M is shown as a function of magnetic field B at T = 2 K (crosses) and T = 300 K (squares) whereas difference of magnetization M_exp_(B, T = 2 K)–M_exp_(B, T = 300 K) is depicted by circles and labeled 2 K–300 K. Black solid lines are fits of Equations (2) and (3) (see, text). Dash lines are eye-guides for M = 0 (emu/g). Lower drawings—magnetization M is shown as a function of temperature T (K) at constant magnetic field B = 0.5 tesla.

The discussion starts with the series of nanopowders prepared in the metal sulfide (MS) system. The representative magnetization data for the freshly measured samples of pre-kesterite and 550 °C-kesterite are shown in Figure 5. In this regard, the results for the 500 °C-kesterite (not shown) are qualitatively alike the 550 °C-kesterite case. The data include (i) magnetization of both materials *versus* magnetic field at T = 2 K and T = 300 K (upper drawings) and (ii) their magnetization *versus* temperature (2–300 K) at constant magnetic field B = 0.5 tesla (lower drawings). The low temperature data in both types of measurements show on overall a typical paramagnetic behavior: magnetization increases monotonously with increasing magnetic field and tends to saturate for the highest fields (Figure 5, upper drawings) whereas during the temperature increase in this range the magnetization decrease is, roughly, proportional to 1/T (Figure 5, lower drawings). At high temperatures, e.g., at 300 K magnetization becomes negative and linear with magnetic field (Figure 5, upper drawings).

**Figure 6:**
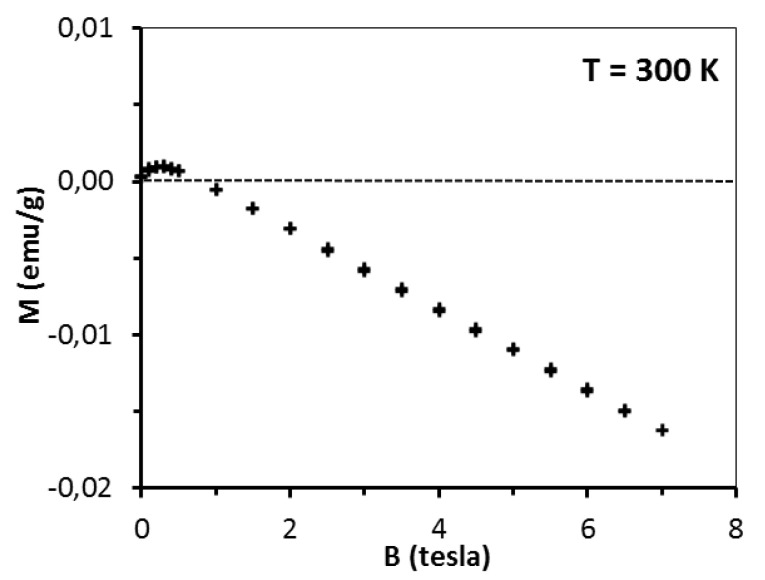
Magnetization M of pre-kesterite from MS system as a function of magnetic field B at temperature T = 300 K. Dash line is eye-guide for M = 0 (emu/g).

This is the effect of surpassing paramagnetism of the sample by diamagnetism of the sample’s lattice. A more detailed insight into the pre-kesterite data, however, points out to a more complex situation in that case. At 300 K, magnetization rises fast at low fields (B < 0.5 tesla) with tendency to saturation and then shows negative linear field dependence (for B > 0.5 tesla) as shown in Figure 6 for pre-kesterite from MS system (expansion of the 300 K-curve in Figure 5).

Since at 300 K both the paramagnetic and diamagnetic components are linear with magnetic field, the low field increase of magnetization suggests an additional component contributing to the measured magnetization. We suppose that in addition to the paramagnetic and diamagnetic components discussed earlier, there is also an extra component in the pre-kesterite nanopowder that has originated from ferro/ferrimagnetic impurities linked to the precursor system or formed during the high energy ball milling (e.g., some likely left-over zinc sulfide ZnS reported to show *d^0^* ferromagnetism [20,21]). We note that such a situation has been widely encountered, for instance, in a series of gallium nitride- and antimonide-based magnetic semiconductors synthesized with magnetic transition metal concentrations close to the solubility limits [38,39,40]. We note that the ferro/ferrimagnetic phase(s) are not observed for any of the kesterite nanopowders in both precursor systems, which suggests that annealing eliminates the phase(s). This is consistent with the presence of some unreacted ZnS in the raw powder containing mostly pre-kesterite, with ZnS being incorporated upon annealing into the evolving tetragonal kesterite structure characteristic as it seems of inherent paramagnetism.

Having the above in mind, the measured magnetic moment of each sample should be regarded as a sum of the paramagnetic component, diamagnetic contribution of the lattice, and possible contribution of unintentional impurities. Therefore, the experimentally measured magnetic moment M_exp_(B,T) can be expressed in the following form of Equation (1).

(1) $$M_{exp}\left(B,T\right)=M\left(B,T\right)+\chi_{dia}*B+C$$

where M(B,T) is the total magnetization of magnetic moments of the pre-kesterite or kesterite component, χ_dia_ is the diamagnetic susceptibility of such component (the property assumed to be temperature-independent in the studied temperature range), and C represents contribution from possible ferro/ferrimagnetic phases. 

As may be noticed from Figure 5 (see, upper row), for both the pre-kesterite and kesterite nanopowders the diamagnetic contributions are appreciable and dominate the measured magnetic moment at relatively high temperatures, e.g., at T = 300 K the measured magnetic moment is negative (diamagnetic). In such a case, precise values of the χ_dia_ and C terms for the sample in question become crucial. Here, proposed is a way to eliminate the (χ_dia_·B + C) contributions by subtracting the high temperature magnetic moment (at T = 300 K, where paramagnetic contribution of magnetic moments is largely quenched) from the low temperature one (at T = 2 K). Assuming that χ_dia_ and C are temperature independent (which is largely true in the temperature range of interest) one arrives at Equation (2).

(2) $$M_{exp}\left(B,T=2K\right)-M_{exp}\left(B,T=300K\right)=M\left(B,T=2K\right)-M\left(B,T+300K\right)$$

Although the nature of the paramagnetic centers is not known, their magnetic moment must result from unpaired electrons (e.g., Cu^2+^ in *d^9^*-configuration, vacancies, surface free radicals, etc.). It is therefore reasonable to assume M(B,T) in the form shown below in Equation (3).

(3) $$M\left(B,T\right)=N*g*\mu_{B}*S*B_{S}\left(B,T\right)$$

where B_S_(B,T) is the Brillouin function [26] for spin S, g = 2.00 is g-factor, μ_B_ is Bohr magneton, and N is the number of spins (magnetic centers) in the sample. Possible interactions between spins can be taken into account by assuming an effective temperature T_eff_ = T − T_0_ instead of experimental temperature T [41,42]. We recall that, formally, T_0_ < 0 corresponds to antiferromagnetic (AFM) interactions whereas T_0_ > 0 means ferromagnetic (FM) interactions.

The results of fitting the experimental data with Equations (2) and (3) are shown in Figure 5 (upper drawings, solid lines). For all the samples the best match is obtained for S = 1/2 and T_0_ = 0. Spin S = 1/2 suggests that the magnetic moment originates from singly occupied/unoccupied orbitals (e.g., *d^9^* configuration). On the other hand, T_0_ = 0 indicates the absence of exchange interaction between magnetic moments. This is not surprising given the concentration of magnetic moments in all samples is very low, i.e., we estimate that the average magnetic moment per one Cu_2_ZnSnS_4_ molecule is 0.014 μ_B_, which corresponds to one magnetic moment generated by spin S = 1/2 per some 70 kesterite molecules. It is worthwhile to note that the estimations provide with the number of magnetic moments in the pre-kesterite, 500 °C-kesterite, and 550 °C-kesterite nanopowders decreasing with annealing according to the ratio of 1:0.86:0.83, respectively. The largest decrease takes place on conversion of pre-kesterite to kesterite whereas the higher annealing temperature has a relatively smaller impact as supported by comparison of both kesterite nanopowders.

**Figure 7:**
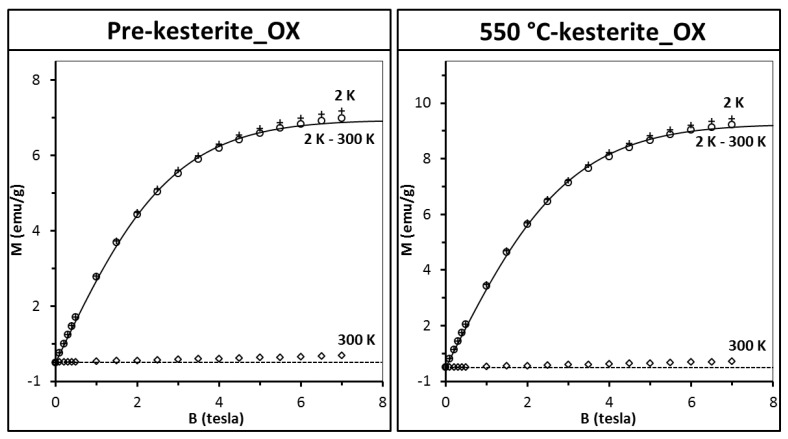
Magnetization M of 3 month-conditioned in air nanopowders (left, pre-kesterite_OX; right, 550 °C-kesterite_OX) from the metal sulfide (MS) system. Magnetization is shown as a function of magnetic field B at T = 2 K (crosses) and T = 300 K (squares). Difference of magnetization M_exp_(B, T = 2 K) – M_exp_(B, T = 300 K) is depicted by circles and labeled 2 K-300 K. Solid lines are fits of Equation (4) (see, text). Dash lines are eye-guides for M = 0 (emu/g).

In order to check the nanopowders susceptibility to oxidation, the samples were conditioned in ambient air for 3 months and then measured again (it should be stressed that the experiment was performed on the very same samples that were originally measured as fresh, see Figure 5). The results for two of the oxidized samples are presented in Figure 7.

The most striking observation is the large increase of magnetization of some 30 times as compared to the original fresh samples (compare with Figure 5, upper drawings). Apparently, conditioning in air results in substantial increase of a number of paramagnetic centers in both pre-kesterite and kesterite. Data analysis that was carried out as previously (Equations (2) and (3), S = 1/2, T_0_ = 0.0) shows that the number of paramagnetic centers increases now 37 times for pre-kesterite, 65 times for 500 °C-kesterite, and 60 times for 550 °C-kesterite. In consequence, magnetization at high temperatures (e.g., 300 K) is positive as paramagnetic contribution prevails over the diamagnetism of the sample’s lattice. Such a large increase of the number of magnetic centers to, formally, about 1 center per 2 molecules could yield pronounced effects of spin–spin exchange interactions since the distance between magnetic centers shortened substantially. In particular, the spin–spin interaction should manifest itself in non-zero effective temperature T_0_ mentioned above. However, no such effects are observed and the best fits are obtained for T_0_ = 0 with accuracy better than 0.1 K.

**Figure 8:**
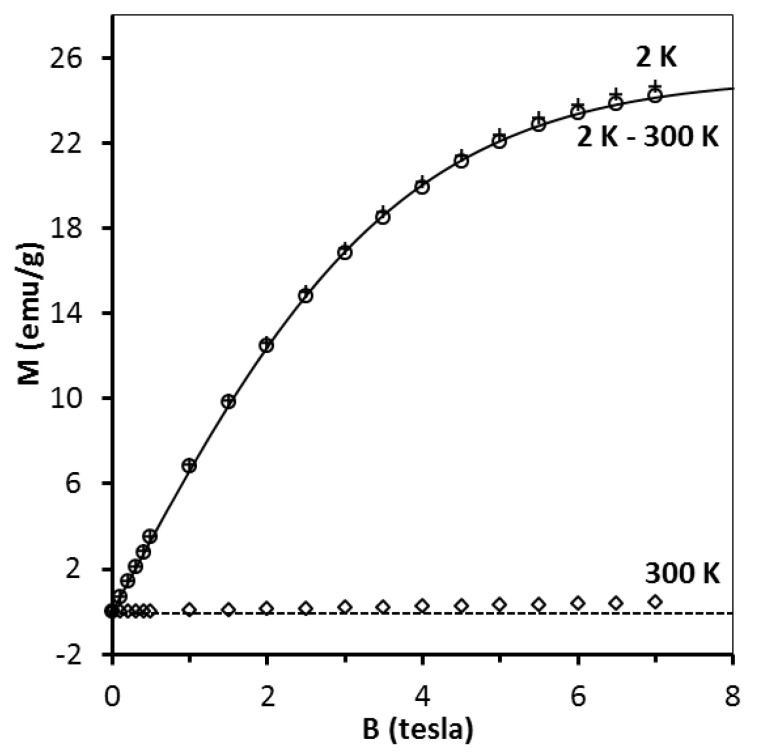
Magnetization M of CuSO_4_·5H_2_O as function of magnetic field B at T = 2 K (crosses) and T = 300 K (squares). Difference of magnetization M_exp_(B,T = 2 K)–M_exp_(B,T = 300 K) is depicted by circles and labeled 2 K–300 K. Solid line is a fit of Equations (2) and (3) for S = 1/2 and T_0_ = −0.52 K (see, text). Dash line is eye-guide for M = 0 (emu/g).

The XRD patterns for all three conditioned in air nanopowders (not shown), i.e., pre-kesterite_OX, 500 °C-kesterite_OX, and 550 °C-kesterite_OX confirm extensive oxidation processes with formation of CuSO_4_·5H_2_O, ZnSO_4_·xH_2_O, SnSO_4_, and with remaining pre-kesterite/kesterite component at the levels of 52 wt.% (pre-kesterite_OX), 61 wt.% (500 °C-kesterite_OX), and 65 wt.% (550 °C-kesterite_OX). Since one of the natural candidates for paramagnetic centers is copper ion Cu^2+^ (configuration *d^9^* and spin S = 1/2), magnetization of pure reference CuSO_4_·5H_2_O powder was measured (Figure 8). It appears that in order to obtain proper description of the data the effective temperature T_eff_ must be used with T_0_ = −0.52 K. Such a T_0_ value indicates antiferromagnetic interaction between Cu^2+^ ions and is in reasonable agreement with the reported Curie–Weiss temperature [43].

Attempts to fit the pre-kesterite and kesterite data (for the fresh and conditioned in air samples) by the CuSO_4_·5H_2_O magnetization curve (i.e., Equation (3) with S = 1/2 and T_0_ = −0.52 K) fail as curvature of the pre-kesterite/kesterite magnetization is noticeably different from that of CuSO_4_·5H_2_O. In fact, a better although not perfect fit is provided by a simple Brillouin function with T_0_ = 0. One must conclude then that the increase of paramagnetism of the samples exposed to air cannot result solely from the formation of CuSO_4_·5H_2_O. However, assuming CuSO_4_·5H_2_O was formed during sample conditioning in air, magnetization should be described by the following function (Equation (4)) where N_1_ (corresponding to pre-kesterite/kesterite) and N_2_ (corresponding to CuSO_4_·5H_2_O) are the only fitting parameters.

(4) $$\begin{array}{cc} M_{exp}\left(B,\;T=2K\right)- & M_{exp}\left(B,\;T=300K\right)\\ & =N_{1}*g*\left[B_{S=\frac{1}{2}}\left(B,\;T=2K,\;T_{0}=0.00K\right)-B_{S=\frac{1}{2}}\left(B,\;T=300K,\;T_{0}=0.00K\right)\right]\\ & +N_{2}*g*\mu_{B}\\ & *\left[B_{S=\frac{1}{2}}\left(B,\;T=2K,\;T_{0}=-0.52K\right)-B_{S=\frac{1}{2}}\left(B,\;T=300K,\;T_{0}=-0.52K\right)\right] \end{array}$$

The resulting fits are shown as solid lines in Figure 7. According to the calculated values of N_1_ and N_2_, in the case of the oxidized 500 °C-kesterite some 19% of magnetization originates from CuSO_4_·5H_2_O whereas for the oxidized 550 °C-kesterite it amounts to 11%. It should be noted that the contents of CuSO_4_·5H_2_O in all fresh samples from the MS system are negligible (the best fit with Equation (4) yields N_2_ parameter equal zero) and also the compound is not detected there by any of the analytical methods applied for materials characterization. We suppose that the kesterite oxidation process and, specifically, Cu^1+^-ion oxidation proceeds through various intermediates such as oxidized sulfur sites to lead to -Cu-S-O species that at some point can result in Cu^2+^-ions on the surface of a sufficiently deep-oxidized nanocrystallite. This is a stage when the secondary magnetic Cu^2+^ centers appear in the kesterite structure, which contribute to the large increase of paramagnetism upon prolonged oxidation. Continued oxidation will at some point lead to the evolution of sulfate SO_4_^2−^ groups and precipitation of the metal sulfates that may pick up water molecules from humid air to form the hydrated salts. In this regard, the XPS S 2p spectra confirm the S–O bonds present in all fresh samples of pre-kesterites and kesterites that were stored in a desiccator and briefly exposed to air during handling thus supporting favored sulfur site oxidation.

**Figure 9:**
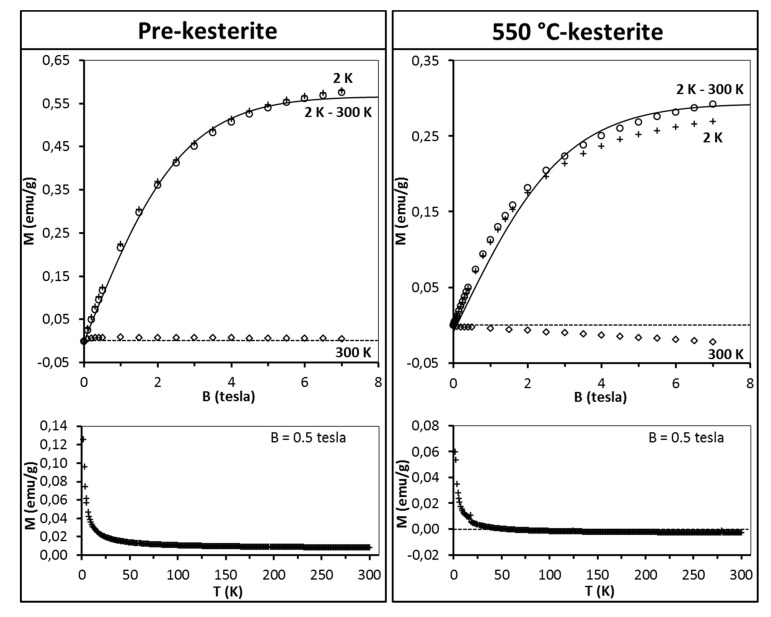
Magnetization M of pre-kesterite (left) and 550 °C-kesterite nanopowders (right) from constituent elements (CE) system. Upper drawings–magnetization M is shown as a function of magnetic field B at T = 2 K (crosses) and T = 300 K (squares) whereas difference of magnetization M_exp_(B,T = 2 K)–M_exp_(B,T = 300 K) is depicted by circles and labeled 2 K–300 K. Black solid lines are fits of Equations (2) and (3) (see, text). Lower drawings–magnetization M is shown as a function of temperature T (K) at constant magnetic field B = 0.5 tesla. Dash lines are eye-guides for M = 0 (emu/g).

Magnetic behavior of the freshly measured materials from the second investigated precursor system, i.e., CE system is similar overall to the MS system as exemplified in Figure 9 for the pre-kesterite and 550 °C-kesterite (compare with Figure 5). It is worth to note that in this system the number of magnetic centers (assuming spin S = 1/2) in the pre-kesterite is found to be about three times larger than it is for the pre-kesterite from the MS system (compare Figure 5 and Figure 9). Moreover, the amount of the ferromagnetic-type phase is also much larger here to the extent that magnetization at 300 K is positive (Figure 9, left lower drawing)—the high temperature asymptotic value of M (T) is positive and much larger (compare Figure 5 and Figure 9). Annealing of the pre-kesterite results in decrease of magnetic centers in the resulting 500 °C-kesterite and 550 °C-kesterite similarly as in the MS system. Also, the initially present ferromagnetic-type phase apparently disappears: magnetization of the 550 °C-kesterite at 300 K becomes negative and linear with magnetic field (Figure 9, right lower drawing)—the high temperature asymptotic value of M (T) is negative. Such behavior is expected in the case of dominant diamagnetic contribution to sample’s magnetic moment and is also observed for the MS system, as discussed earlier. Similarly as for the freshly measured materials in the MS system, no traces of CuSO_4_ are detected for the equivalent cases in the CE system during fitting the data (the best fits with Equation (4) were obtained for N_2_ = 0).

## 4. Summary and Conclusions

The results of the magnetic part of study can be summarized as follows. All freshly measured pre-kesterite and kesterite nanopowder samples from both precursor systems contain paramagnetic centers that can be successfully described by spin S = 1/2 while attempts to use different spin values provide noticeably worse description of magnetization. Concentration of magnetic centers is roughly 1 center per about 70 pre-kesterite/kesterite molecules (in other words, one molecule bears 1/70 spin S = 1/2) for MS system and 1 center per about 24 of such molecules for CE system. The most probable candidates for such centers are Cu^2+^ ions that can be broadly said to have originated from oxidation of Cu^1+^ ions in the kesterite species, substituted Zn^2+^-ions by Cu^2+^-ions in copper rich kesterite, broken bonds at the grains surface (free radicals) or vacancies with spin S = 1/2.

A 3-month conditioning in ambient air of the MS samples results in large increase of the number of paramagnetic centers, i.e., by nearly two orders in magnitude corresponding to changing the number from roughly 1 center per about 70 molecules to 1 center per about 2 molecules. The conditioning is associated with pronounced nanopowder oxidation. In the case of oxidized samples, some 10–20% of the increase of magnetization can be ascribed to the formation of CuSO_4_·5H_2_O of which presence is independently confirmed by XRD. On the other hand, contents of CuSO_4_·5H_2_O in freshly measured nanopowders from both the MS and CE systems seem to be negligible, if any.

Although in principle it is possible to ascribe weak paramagnetism for the freshly measured samples to broken bonds at particle surfaces or specific site vacancies via *d^0^* paramagnetism, the observed increase of paramagnetism for the samples conditioned in air (roughly one broken bond per every second molecule!) cannot be accounted for this way. In this regard, for broken bonds and site vacancies one should rather expect reduction of their number with prolonged exposition to air and resulting drop of magnetization. Since the experimental observations are just opposite, accordingly, we tend to rule out such paramagnetic centers.

In our opinion the most probable magnetic species responsible for the observed paramagnetism are Cu^2+^ centers. They appear to be present both in the pre-kesterite and annealed kesterite nanopowders in clearly higher amounts in the former. This is true for both precursor systems although their amount in the constituent element (CE) system is roughly three times larger. We suppose that the primary Cu^2+^ centers in the freshly measured samples originate from adventitious oxidation during the two-step synthesis of kesterite (high energy ball milling and annealing) by oxygen introduced mainly as metal oxide passivation layers in powder substrates and by not strictly adhering to anaerobic conditions (brief exposure to air/oxygen and moisture during handling the pre-kesterite and kesterite nanopowders). In this regard, our recent preliminary direct determinations of oxygen content in these materials indicate possible significant O-contents and such studies are to be extended for kesterite materials in a systematic way. It is also worth to point out that the O-contents in the precursor metals (constituent element system) appear to be overall higher than in the precursor metal sulfides (metal sulfide system) and this relates well to much higher paramagnetism in the products from the former. This points out to some preventing measures to be taken to minimize the primary oxygen contents in the precursors such as temperature treatment under hydrogen flow or applications of much higher excess of sulfur as an oxygen scavenger at high processing temperatures. As far as the kesterite conditioning in ambient air is concerned, it is associated with a relatively slow (days to weeks) continuous oxidation that at some point results in the metal sulfates (hydrates) but proceeds via the formation of Cu^2+^ centers likely on crystallite surfaces. These secondary Cu^2+^ magnetic centers contribute to significantly increased paramagnetism of the 3-month conditioned kesterite samples.

The failure to detect the primary Cu^2+^ centers by XPS measurements may likely result from a relatively low sensitivity of the method (detection limits of standard measurements are in one per thousand atoms range) and the closeness of the Cu^2+^ energy peaks to the prevailing Cu^1+^ energy peaks.

A fascinating and still difficult to approach in a quantitative way interrelation of kesterite magnetism and effectiveness of NMR resonance (*vide supra*) appears to find some important justifications. First, the fact that the pre-kesterite nanopowders are NMR defunct suggests a presence of a collective magnetic field such as characteristic for ferromagnetism. The confirmed ferromagnetic component in the magnetization of pre-kesterite coupled with paramagnetic contributions from the Cu^2+^ centers could, therefore, be responsible for it. The emergence of the ^65^Cu and ^119^Sn MAS NMR signals upon annealing coincides with disappearance of ferromagnetism and relative decrease of paramagnetism in the resulting kesterite. Second, the varying intensities of both the ^65^Cu and ^119^Sn MAS NMR peaks in the kesterite nanopowders, i.e., higher for the 550 °C-kesterite than for the 500 °C-kesterite, are consistent with lower paramagnetism of the former. Since paramagnetism of the Cu^2+^ centers has a local character and exerts a local impact in the structure it may, therefore, inactivate from NMR-induced resonance only a certain proportion of the nuclei and what is important – both of copper and tin as observed. What introduces complications in a quantitative appraisal of the phenomenon is a lack of knowledge on the distribution of the paramagnetic Cu^2+^ centers including likely differences between the surface layers and the core of kesterite crystallites of varying sizes. Nevertheless, normalized NMR determinations may be invaluable in providing supplementary data on kesterite magnetic properties.

All the major observations support a notion that the unrecognized aspect of kesterite oxidation in the synthesis and upon storage in ambient air requires a deeper insight with measurements of magnetic properties possibly revealing specifics and extent of oxidation so the practical application potentials of kesterite.
